# A novel finding of intra-genus inhibition of quorum sensing in *Vibrio* bacteria

**DOI:** 10.1038/s41598-022-19424-w

**Published:** 2022-09-08

**Authors:** Huong Thanh Hoang, Thuy Thu Thi Nguyen, Ha Minh Do, Thao Kim Nu Nguyen, Hai The Pham

**Affiliations:** 1 Research group for Physiology and Applications of Microorganisms (PHAM group), GREENLAB, Center for Life Science Research (CELIFE), Faculty of Biology, University of Science – Vietnam National University in Hanoi, 334 Nguyen Trai, Thanh Xuan, Hanoi, Vietnam; 2 Department of Physiology and Human Biology, Faculty of Biology, University of Science – Vietnam National University in Hanoi, 334 Nguyen Trai, Thanh Xuan, Hanoi, Vietnam; 3 Department of Cell Biology, Faculty of Biology, University of Science – Vietnam National University in Hanoi, 334 Nguyen Trai, Thanh Xuan, Hanoi, Vietnam; 4 Department of Microbiology, Faculty of Biology, University of Science – Vietnam National University in Hanoi, 334 Nguyen Trai, Thanh Xuan, Hanoi, Vietnam; 5 Present Address: Cell Therapy Department, Hi-Tech Center, Vinmec International General Hospital Joint Stock Company, Times City, 458 Minh Khai, Hai Ba Trung, Hanoi, Vietnam

**Keywords:** Bacteriology, Bacteria

## Abstract

Quorum sensing is the process by which microbial cells sense and respond to the co-presence of others in their surrounding, through the detection of their autoinducers associated with gene expression regulation and thereby controlling many physiological processes, such as biofilm formation and/or bioluminescence, etc. In *Vibrio* bacteria, where quorum sensing is relatively well understood with three commonly known autoinducers (HAI-1, AI-2 and CAI-1), both intra-species and inter-species cell–cell communications occur but no inter-*Vibrio*-species quorum sensing inhibition has been reported. In this study, by screening bacterial isolated from soil and mud samples in a northern province in Vietnam, we discovered a strain that reduced more than 75% of the bioluminescence of a *Vibrio harveyi*, with evidence showing that such an inhibition might be associated with quorum sensing inhibition. The strain, designated as XTS1.2.9, was identified to be a *Vibrio parahaemolyticus* bacterium based on its morphological, physiological, biochemical and phylogenetic characteristics. We also tested XTS1.2.9 for its bioluminescence inhibition against different mutants lacking different quorum sensing autoinducers by using plate assays. The results showed that XTS1.2.9 inhibited the bioluminescence of the mutants having sensor 1, especially the one detecting CAI-1, and lacking sensor for AI-2; while it did not inhibit the mutants having only sensor for AI-2 and lacking sensor 1. Therefore, we propose an intra-genus quorum sensing inhibition mechanism involving CAI-1 to explain for such interactions between *Vibrio parahaemolyticus* and *Vibrio harveyi*. This phenomenon is reported for the first time and may have certain scientific and application implications.

## Introduction

Quorum sensing (QS) is the process by which microbial cells (mostly bacteria) sense and respond to the co-presence of other microbial cells in their surrounding, through gene expression regulation^1^. The sensing process is solely modulated through small signal molecules called as autoinducers (AIs) that are self-produced and released by quorum sensing bacteria^1^. Thus, the change in the concentrations of autoinducers can reflect the change in cell density. When an autoinducer is concentrated up to a certain level, the so-called minimal threshold stimulatory concentration, the expression of one or many genes can be activated or inactivated. Bacteria can use this mechanism to regulate various physiological processes such as biofilm formation, virulence, motility, antibiotic production, sporulation, etc.^1,2^. Therefore, in general, quorum sensing enables bacteria to communicate with one another and to coordinate their gene expressions, though which coordinating their behaviors at population level or even community level.

Quorum sensing is common among bacteria, including the *Vibrio* species, many of which have been used as models to unveil more about this unique phenomenon^1,3^. *V. cholera, V. harveyi and V. parahaemolyticus* are among the vibrios that have been studied the most in terms of quorum sensing (probably because they are noticeable pathogens) and thus their quorum sensing mechanisms seem to have been understood the most^3–8^. Accordingly, in vibrios, three quorum sensing systems have been reported, i.e. quorum sensing can be modulated through 3 different kinds of autoinducers. System 1 involves an acylated homoserine lactone (AHL) autoinducer (3-hydroxy-C_4_-homoserine lactone, denoted as HAI-1) and its respective sensor (denoted as the HAI-1 sensor), which is common to not only vibrios but also most bacteria^6,9^. System 2 consists of a boron-containing protein based autoinducer, called AI-2, and the AI-2 sensor^1,10^. This system is also considered to occur globally in a wide range of both Gram-positive and Gram-negative bacteria^4^. Another system, which is actually a typical system 1 in *V. cholerae*, involves the so-called CAI-1 autoinducer and its respective sensor^4–6^. This CAI-1 system is the third system in *V. harveyi, V. parahaemolyticus* and several other vibrios^4,6,7^. The aforementioned quorum sensing systems work in harmony and are regulated in a relatively universal process to ultimately control several important physiological processes in *Vibrio* species, such as biofilm formation and swarming (as in *V. cholerae* and *V. parahaemolyticus*)^3,5,8^ and/or bioluminescence (as in *V. harveyi*)^6^, etc. Therefore, disruptions in quorum sensing in these *Vibrio* bacteria can result in disruptions in the associated physiological processes, such as reduced virulence as in *V. cholerae* and *V. parahaemolyticus* or quenched bioluminescence in *V. harveyi*^11^. Indeed, bioluminescence quenching is considered as a strong indication of quorum sensing disruption (or inhibition) in *V. harveyi*, as demonstrated by a number of studies^1,2,6,11,12^.

The quorum sensing systems described above mostly work for not only intra-species cell–cell communications but also inter-species ones^13^. Within the genus of *Vibrio*, those inter-species quorum sensing interactions do occur and are generally synergistic. For example, *V. cholera* uses its autoinducers (CAI-1 and AI-2) to detect the kin and nonkin cells to control its biofilm formation and dispersal^3^. Another species, *V. vulnificus*, was reported to rely on inter-species AI-2-mediated quorum sensing interactions for its resuscitation from the “viable but non-culturable” state^14^. Also, there was evidence that cross-species quorum sensing interactions between *V. plendidus* and *V. aestuarianus* modulated their metalloprotease gene expression, which is essential in their pathogenicity^15^. The aforementioned interactions are all “positive” although inter-species cross-talks in bacteria may also result in antagonisms^13^. However, up to our knowledge, there has been no report on inter-species or intra-genus quorum sensing inhibitions among *Vibrio* species. In this study, we describe for the first time a quorum-sensing-associated inhibition to a *Vibrio* species upon its contact with another *Vibrio* species. We also tried to explore the mechanism behind the inhibition and proposed some implications of this finding.

## Results

### Possible quorum-sensing inhibitory activity of strain XTS1.2.9 against the tested *V. harveyi* strain

From 16 soil and mud samples collected in some northern provinces in Vietnam, 381 isolates were obtained growing on LB medium containing 3% NaCl. We subsequently screened them for those exhibiting the inhibition of the QS-associated bioluminescence of *Vibrio harveyi* NBRC15634 (or *V.h* in short), by using a 96-well plate assay devised by our group. Several strains exhibited the inhibition (data not shown) and the one expressing the most significant inhibitory activity was XTS1.2.9, designated according to the group’s code.

**Figure 1:**
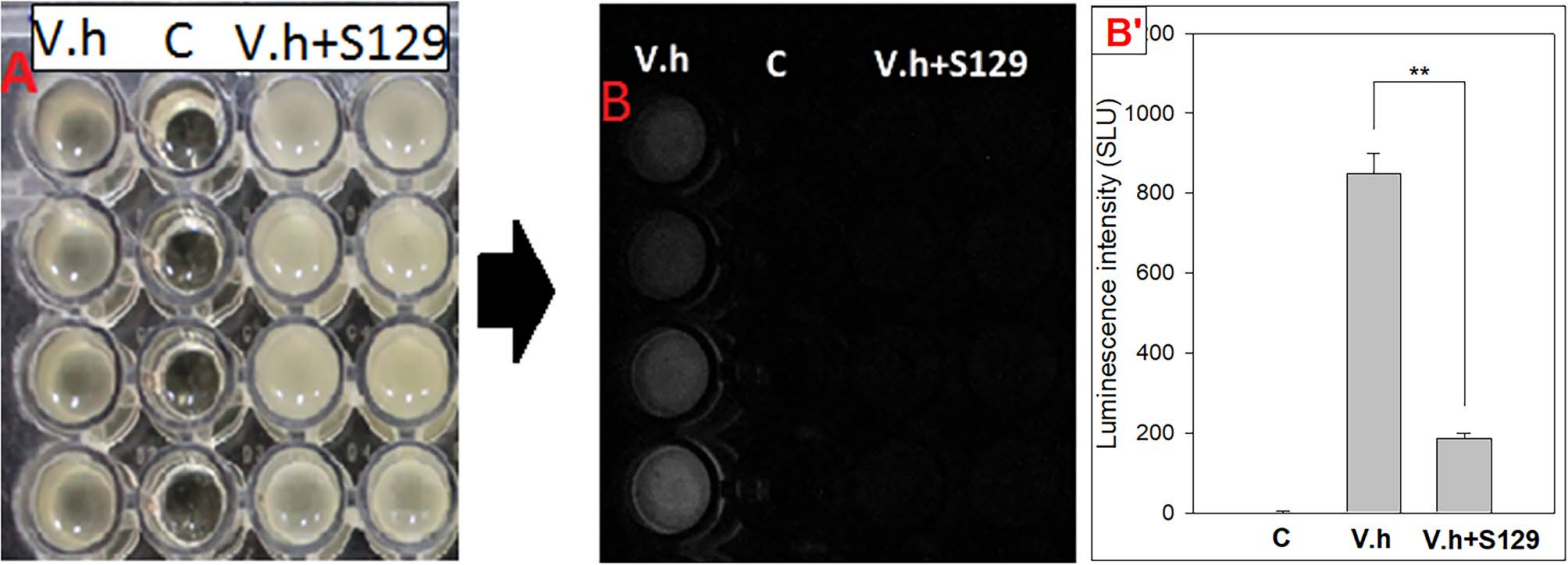
96-well plate assay showing the QS-associated bioluminescence inhibitory activity of strain XTS1.2.9 against the tested *V. harveyi* strain. (**A**) plate wells observed under normal light; (**B**) plate wells observed in the dark; (**B’**) luminescence from the wells measured by luminometer. Notes: V.h: wells each containing 240 µl of LB broth with 3% NaCl and 10 µl of *V. h* culture broth (OD_600nm_ ~ 1.0); C: control wells each containing 250 µl of sterilized 3% NaCl LB broth only; V.h + S129: wells each containing 240 µl of sterile LB broth with 3% NaCl and 10 µl of *V. h* culture broth (OD_600nm_ ~ 1.0) added with 1 µl of XTS1.2.9 culture broth (in LB, OD_600nm_ ~ 1.0)*.* **indicates statistically significant difference (*p* < 0.005).

Strain XTS1.2.9 reduced more than 75% the bioluminescence of *V.h* when the wells containing the *V.h* culture (in LB broth) were supplemented with the XTS1.2.9 culture (in LB broth) (Fig. 1). To investigate whether the inhibitory activity of XTS1.2.9 was due to growth competition or due to the products in its culture, we carried out similar assays by mixing the culture broth of *V.h* with: (i) the same volume of the lysed-cell culture broth of XTS1.2.9, (ii) the same volume of the supernatant of the centrifuged culture broth of XTS1.2.9, (iii) the cell pellet of the centrifuged culture broth of XTS1.2.9, and (iv) different amounts of the cell-free culture broth of XTS1.2.9. The results (Table 1) clearly showed that the XTS1.2.9 culture broths containing few cells or dead cells or even no cells could still cause the bioluminescence reduction while XTS1.2.9 cells removed from the culture broth could only slightly reduced the bioluminescence.

**Table 1:** Bioluminescence of *V.h* in the different 96-well plate assays with XTS1.2.9

Test	With lysed-cell XTS1.2.9 culture broth	With supernatant of centrifuged XTS1.2.9 culture broth	With cell pellet of centrifuged XTS1.2.9 culture broth	With cell-free XTS1.2.9 culture broth					
				5 µl	10 µl	25 µl	50 µl	100 µl	150 µl
Bioluminescence	−	−	( +)	−	−	−	−	−	−

( +): weakly luminescent; −: almost dark (little luminescence could be observed).

**Figure 2:**
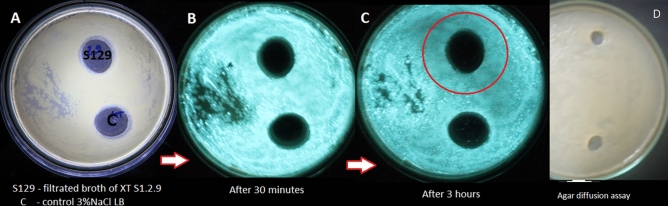
Plate assay showing QS-associated bioluminescence inhibitory activity of strain XTS1.2.9 against the tested *V. harveyi* strain. Notes: (**A**): a plate with *V.h* already growing on it and holes filled with 500 µl of the cell-free XTS1.2.9 culture broth and 500 µl of sterile LB broth with 3% NaCl (control); (**B**): the same plate in the dark 30 min after the additions of the broths; (**C**): the same plate in the dark 3 h after the additions; (**D**): agar diffusion assay showing no growth inhibition against *V.h* by the cell-free XTS1.2.9 culture broth. (The red circle indicates a clearly darker zone observed around the hole filled with the cell-free XTS1.2.9 culture broth. Experiments were done in triplicate showing similar results and only a representative result is shown here)**.**

The inhibitory activity of XTS1.2.9 against the bioluminescence of *V.h* was even more clearly demonstrated by the agar plate assay (Fig. 2). Upon the contact of *V.h* to the cell-free XTS1.2.9 culture broth, the bioluminescence of *V.h* was not affected within 30 min. (Fig. 2B) but was significantly reduced after 3 h, while the control was not (Fig. 2C). In addition, the growth of *V.h* was not inhibited by the cell-free XTS1.2.9 culture broth (Fig. 2D).

Altogether, the results suggest that there are some components produced by XTS1.2.9 that might inhibit the bioluminescence of *V.h*. More interestingly, as the bioluminescence was not quenched quickly, it is possible that those components did not target the luminescent machinery of *V.h* but the physiological processes associated with its bioluminescence, most probably quorum-sensing.

**Figure 3:**
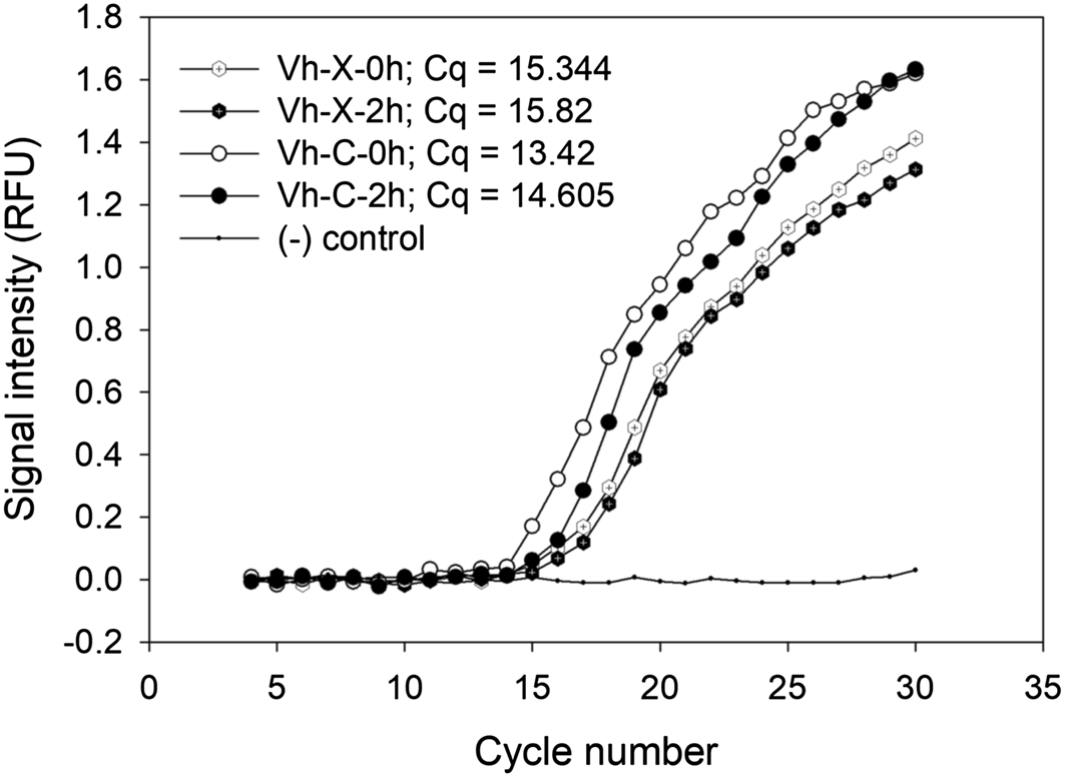
Amplicon dissociation curves of the different RT-PCRs testing the expression levels of *luxR* in 4 different experimental cases. These cases include: (i) the tested *V. h* culture right after being mixed with the cell-free XTS1.2.9 culture broth (Vh-X-0 h); (ii) the tested *V. h* culture two hours after being mixed with the cell-free XTS1.2.9 culture broth (Vh-X-2 h); (iii) the tested *V. h* culture right after being mixed with sterile LB broth containing 3% NaCl (Vh-C-0 h); (iv) the tested *V. h* culture two hours after being mixed with sterile LB broth containing 3% NaCl (Vh-C-2 h). Notes: (−) control: RT-PCR negative control (sterile water). (Experiments were done in triplicate showing similar results and only a representative result is shown here)**.**

We further investigated how the cell-free XTS1.2.9 culture broth might affect the quorum-sensing machinery of *V.h* by using reverse-transcription PCR to evaluate the expression level of *luxR*, the gene encoding for the key quorum-sensing regulator that connects quorum-sensing signaling and bioluminescence^6,16^. Noticeably, immediately when *V.h* was mixed with the cell-free culture broth, the expression level of *luxR* in *V.h* was significantly lower (*p* < 0.05) compared to that in the control (Cq =  ~ 15.3 vs. 13.4) (Figs. 3, S7). After two hours of mixing, the expression level of *luxR* in *V.h* slightly decreased further (Cq =  ~ 15.8) and remained significantly lower (*p* < 0.05) than that in the control (Cq =  ~ 14.6) (Figs. 3, S7). These results are another evidence that some components produced by XTS1.2.9 can interrupt the operation of the quorum-sensing machinery of *V.h*.

### Taxonomic identification showing strain XTS1.2.9 to be a *Vibrio parahaemolyticus* bacterium

Strain XTS1.2.9 was found to be a Gram-negative bacterium, with vibrio-like cell morphology (Fig. S1). This observation was very stimulating and thus we further characterized this strain in terms of its vibrio-related biochemical characteristics (according to Jayasinghe et al.^17^) as well as phylogenetic characteristics. XTS1.2.9 displayed the biochemical characteristics (Table 2) that are typical for *Vibrio parahaemolyticus*, according to Jayasinghe et al.^17^. We further sequenced its 16S rRNA gene and our further analyses discovered that the obtained sequence (GenBank accession number ON150858) was 98.89% homologous to that of *V. parahaemolyticus*. As this might not be supportive enough for the identification of the strain, we continued to analyze the *Vibrio*-specific house-keeping gene *recA*^18^ of the strain and discovered that its sequence (GenBank accession number ON248832) was also 99.40% homologous to that of *V. parahaemolyticus*.

**Table 2:** Summary of key biochemical characteristics of strain XTS1.2.9

Test	Growth on TCBS medium	Growth in 0% NaCl	VP test	MR test	ONPG test
Reaction	Green	−	−	−	−

With all the data stated above, we are affirmative that strain XTS1.2.9 belongs to the species *Vibrio parahaemolyticus* and designate it as *V. parahaemolyticus* XTS1.2.9.

### Mechanism exploration: activities of XTS1.2.9 against some quorum-sensing mutants of *V. harveyi*

**Figure 4:**
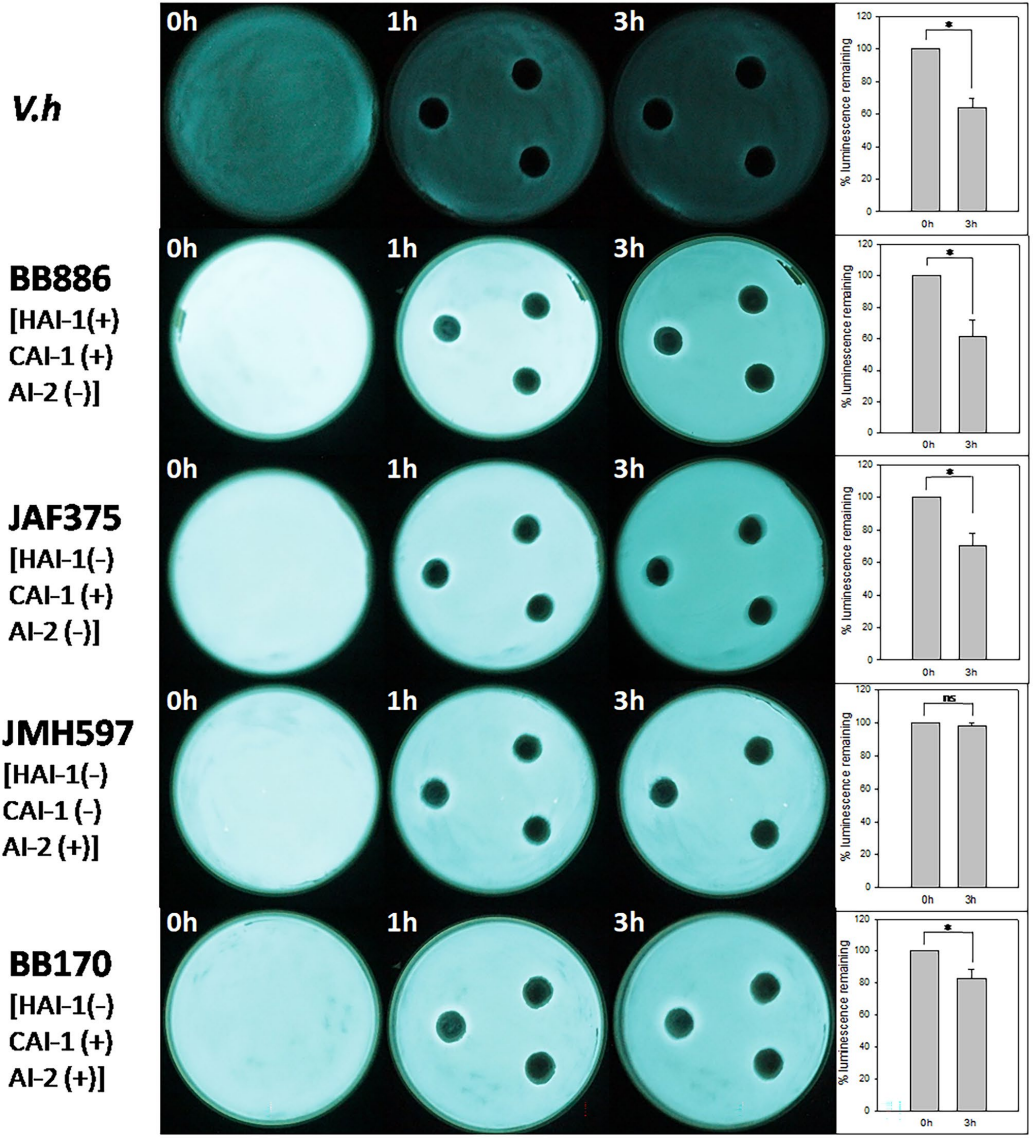
Plate assay results showing QS-associated bioluminescence inhibitory activities of strain XTS1.2.9 against the tested *V. h* strain and different *V. harveyi* mutants deficient in the capabilities of detecting different QS inducers. Notes: The plates contained the strains already growing on LB agar containing 3% NaCl. Each hole in the plate agar was filled with 500 µl of the cell-free XTS1.2.9 culture broth and bioluminescence intensities of the plates were measured with time (after 1 h and 3 h). The bar chart on the right of each row displays the reduction (in percentage) in the intensity of the bioluminescence of the respective strain after 3 h (calculated by using ImageJ to analyze the respective plate images). * indicates a statistically significant difference (*p* < 0.05) between the two datasets in each case, while ns indicates no significant difference (*p* > 0.05). (Experiments were done in triplicate showing similar results and only a representative result is shown here)**.**

In order to understand more about the mechanism behind the inhibition of quorum-sensing associated bioluminescence of *V.h*, *V. parahaemolyticus* XTS1.2.9 was investigated for the inhibitory activities against bioluminescence of several *V. harveyi* mutants deficient in sensing different kinds of autoinducers. Plate assay results (Fig. 4) strongly indicated that the inhibition was almost unchanged for the mutants lacking sensor 2 and having sensor 1 (BB886 and JAF375). Specifically, as calculated by ImageJ (Fig. 4), bioluminescence intensities of these mutants were still reduced about 40% after 3 h they were in contact with the cell-free culture broth of XTS1.2.9. However, the inhibition did not happen with the mutant lacking both sensor HAI-1 and sensor CAI-1 and having only sensor 2 (JMH597) (Fig. 4). Interestingly, the inhibition seemed restored with the mutant lacking sensor HAI-1 but having sensor CAI-1 and sensor 2 (BB170, which is the CAI-1 gene complementation strain to JMH597), although the level of the restored inhibition was not as high as in other cases.

**Figure 5:**
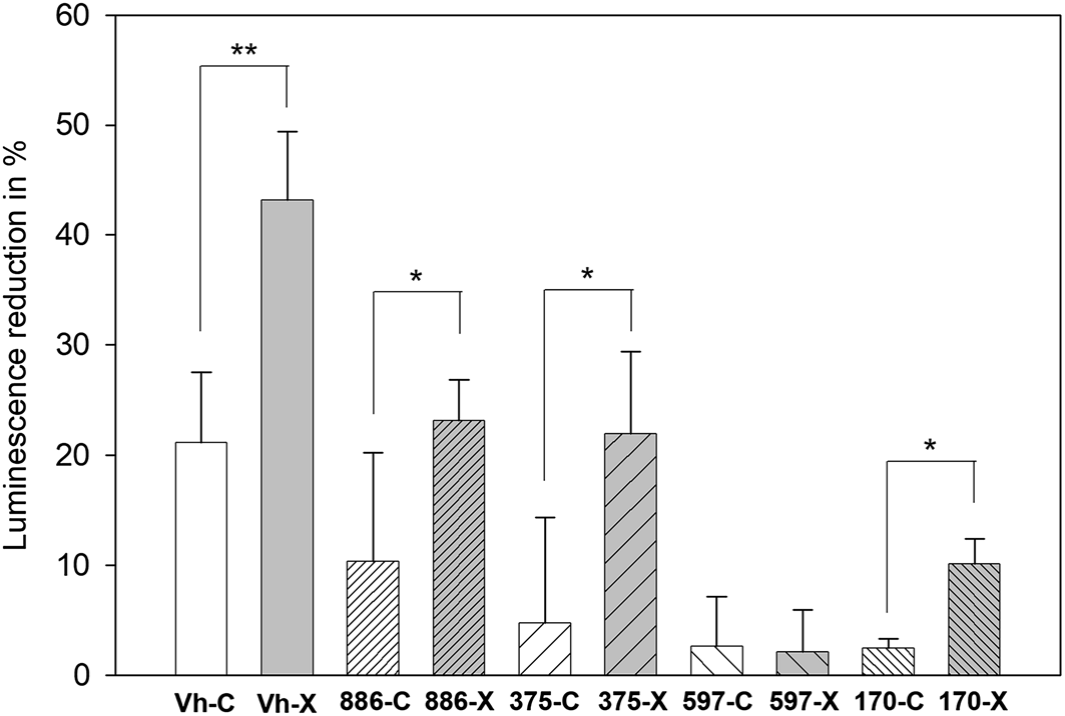
QS-associated bioluminescence reduction (in %) after 3 h when the tested *V. h* strain and the different QS-associated *V. harveyi* mutants were tested with the cell-free XTS1.2.9 culture broth (X) and sterile LB broth containing 3% NaCl (C, the control). Notes: Vh: the tested *V.h* strain (wild type); 886: the BB886 mutant [HAI-1( +), CAI-1 ( +), AI-2 (−)]; 375: the JAF375 mutant [HAI-1(−), CAI-1 ( +), AI-2 (−)]; 597: the JMH597 mutant [HAI-1(−), CAI-1 (−), AI-2 ( +)]; 170: the BB170 mutant [HAI-1(−), CAI-1 ( +), AI-2 ( +)]. Luminescence signals were measured with a luminometer under the same conditions. Statistically significant difference between each (X) dataset and its respective (C) dataset was indicated as **p < 0.005 or *p < 0.05.

As picture-based calculations may be not sufficiently reliable, we further investigated the inhibitory effects of XTS1.2.9 on bioluminescence of the mutants by measuring luminescence signals from the assay plates using a luminometer. The results (Fig. 5) were relatively consistent with the picture-based analysis results (Fig. 4). Specifically, treating with the cell-free XTS1.2.9 culture broth could reduce the bioluminescence of *V.h*, BB886 (sensor 1 ( +); sensor 2 (−)) and JAF375 (sensor HAI-1 (−); sensor CAI-1 ( +); sensor AI-2 (−)) by approximately 22%, 13% and 17% more than the control. On the other hand, such a treatment did not seem to have any different effect on the bioluminescence of JMH597 (having only sensor 2), in comparison with the control (no treatment). However, the treatment exerted a slight but significant inhibitory effect on BB170, which is the CAI-1 complementation strain to JHM597^19^, by reducing its bioluminescence about 8% more than the control (*p* < 0.05). These results confirm that sensor 1 (particularly sensor CAI-1) might be the component that is sensitive to the cell-free XTS1.2.9 culture broth while sensor 2 might not.

## Discussion

### Possible mechanism of the inhibitory effect on quorum-sensing of *V.h* by *V. parahaemolyticus* XTS1.2.9

As described above, the inhibition of quorum sensing in *V.h* by XTS1.2.9 could be demonstrated by the inhibition of bioluminescence of *V.h*. This is strongly supported by the fact that the bioluminescence quenching did not occur quickly but occurred only after 3 h upon the contact of *V.h* with the cell-free XTS1.2.9 culture broth. Indeed, luminescence is strongly associated with quorum sensing and many studies reported that changes in the former reflected changes in the latter^20,21^. Moreover, our RT-PCR results (Fig. 3) also suggest that such *V.h*-XTS1.2.9 contact seems to immediately reduce the expression of *luxR*, the gene encoding a key quorum-sensing regulator in *V.harveyi*^16^. According to Henke et al.^6^, LuxR also regulates bioluminescence and thus a decrease in *luxR* expression level means reduced bioluminescence, which matches the other observations. However, it seems that it takes some time (e.g. at least > 30 min.) for bioluminescence reduction to be effective after the expression of *luxR* is reduced, probably due to the cells that are already luminescent. Furthermore, our results also demonstrated that the different quorum sensing *V.h* mutants exerted different bioluminescent responses to the cell-free XTS1.2.9 culture broth. Therefore, the above-mentioned evidences altogether support the hypothesis that certain components produced by XTS1.2.9 can inhibit quorum sensing in *Vibrio harveyi*, resulting in its bioluminescence reduction.

**Figure 6:**
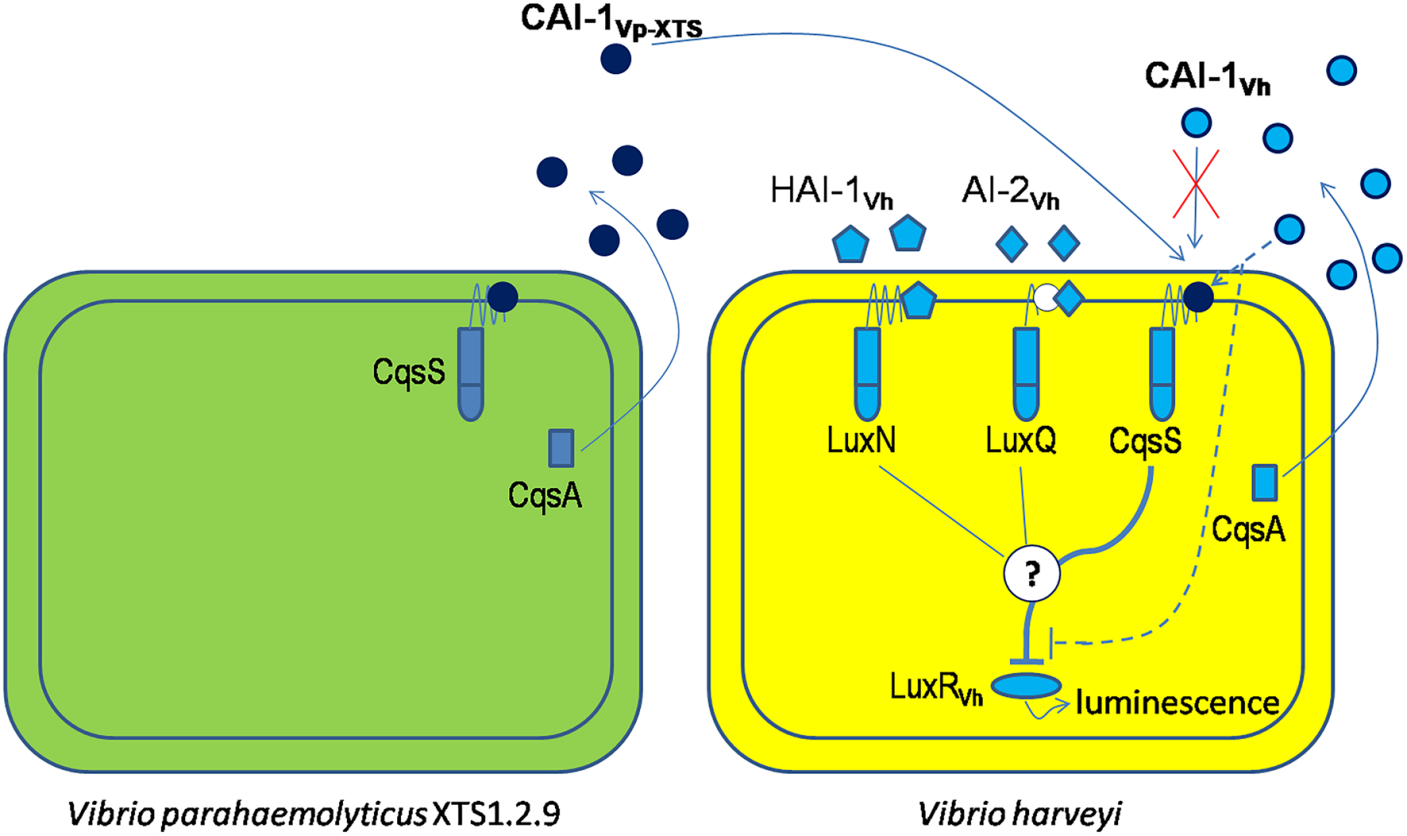
Hypothetical mechanism of quorum sensing inhibition of *Vibrio harveyi* caused by the autoinducer CAI-1 produced by *Vibrio parahaemolyticus* XTS1.2.9. Notes: CAI-1_Vp-XTS_: CAI-1 produced by XTS1.2.9; CAI-1_Vh_, HAI-1_Vh_ and AI-2_Vh_: the autoinducers CAI-1, HAI-1 and AI-2, respectively, produced by *V.h*; LuxN, LuxQ and CqsS: the respective sensors for the autoinducers HAI-1, AI-2 and CAI-1; LuxR_Vh_: the key quorum-sensing response regulator in *V. harveyi*. The antagonistic binding of CAI-1_Vp-XTS_ seems to cause the inhibition of the expression of LuxR_Vh_ by an unknown signaling process (indicated by the question mark) that may surpass those caused by HAI-1 and AI-2. The dashed lines imply what would happen (LuxR_Vh_ expression would not be inhibited) if CAI-1_Vh_ was not blocked and could bind to its cognate sensor CAI-1 (CqsS) of *V.h*.

The inhibitory effect of XTS1.2.9 on quorum sensing in *Vibrio harveyi* might be through targeting its quorum-sensing sensor 1, especially sensor CAI-1, and not through its quorum-sensing sensor 2. This is strongly supported by the results that the mutants lacking sensor 2 (BB886 having sensor 1 and JAF375 having only sensor CAI-1) still displayed bioluminescence reduction when in contact with the cell-free XTS1.2.9 cell broth, while the mutant having sensor 2 and lacking sensor 1 (JMH597) did not. Furthermore, the CAI-1-complemented mutant (BB170) of the latter seemed to restore such a behavior. We should note that both *V. harveyi* and *V. parahaemolyticus* have the CAI-1-modulated quorum sensing system (the CqsA/CqsS system)^6,7^. Moreover, CAI-1 is known to function as a signal for inter-vibrio cell–cell communication^6^, although AI-2 is believed to be a more global inter-species quorum sensing signal^3^. Notably, there has not been any report about the quorum-sensing-inhibiting activity associated with CAI-1 between two *Vibrio* species. Therefore, in this study, the finding that the inter-species quorum-sensing inhibitory interaction between a *V. harveyi* and a *V. parahaemolyticus* might be executed through CAI-1 is surprising. Indeed, *V. harveyi* is known to produce CAI-1 that is similar to that produced by *V. cholera*, which is (*S*)-3-hydroxytridecan-4-one^5^, while *V. parahaemolyticus* produces the so-called CAI-1_Vp_ (3-hydroxyundecan-4-one)^7^, which is 28 (-(CH_2_)_2_-) less than those of CAI-1. We hypothesize that the CAI-1 produced by XTS1.2.9 (CAI-1_Vp-xts_) might be even more varied somehow, which might not work well as a signal molecule for *V. harveyi* (an agonist) but instead as an antagonist for *V. harveyi* CAI-1 (CAI-1_Vh_). This is actually possible because there has been evidence that signal molecule analogs could act as antagonists instead of agonists^22^. This phenomenon is very similar to the inhibitions of quorum-sensing regulated bioluminescence caused by some natural antagonists^11^, except that the antagonist here is astoundingly produced by another *Vibrio* species. Our hypothesis (Fig. 6) is that CAI-1_Vp-xts_ might bind to sensor CAI-1 of *V.h* and thus blocking CAI-1_Vh_ from binding to the sensor. The binding of CAI-1_Vp-xts_ might not provoke the same signaling response as CAI-1_Vh_ does and therefore the expression of *luxR* decreases (as demonstrated by our RT-PCR results—Fig. 3), leading to reduced bioluminescence. Such antagonistic inhibition might be so predominant that it can drown the signaling responses caused by HAI-1 and AI-2. As we could not verify such hypothetical mechanism within the scope of this study, it should be explored in further studies.

### Possible implications of intra-genus QS inhibition among the *Vibrio* species

Intra-genus quorum sensing interactions do occur among different *Vibrio* species but are known to be synergistic, i.e. they involve the shared use of signal molecules to modulate common physiological processes such as metalloprotease gene expression, resuscitation and/or controlling biofilm formation and dispersal^3,14,15^. Moreover, these interactions solely involve the global autoinducer AI-2. Indeed, inter-species cross-talks are common in bacteria and may also result in antagonisms^13^. However, there has been no report on intra-genus quorum sensing inhibitions among *Vibrio* species, such as the one in this study. The inhibition may be associated to the competition between some *V. parahaemolyticus* and *V. harveyi* for habitats and substrates. These two species are both known to be parasitic and pathogenic to marine animals, although in general their niches and substrates do not seem to be overlapping^23,24^. Moreover, no cross-inhibition between them has been reported. Thus the reason for the quorum sensing inhibition on *V. harveyi* caused by *V. parahaemolyticus* XTS1.2.9 remains a question. It is also unclear why such an inhibition involves CAI-1, while the CAI-1 molecules from both species seem similar. It is possible that XTS1.2.9 is only a natural mutant that happens to randomly produce a variant of CAI-1 that can be antagonistic to that of *V. harveyi*. If so, XTS1.2.9 can be an interesting tool to study the CAI-1-associated cell–cell inter-species communications in vibrios.

The inhibitory activity of *V. parahaemolyticus* XTS1.2.9 on the quorum sensing of *V. harveyi* may offer an approach to control *V. harveyi* in the situations where *V. parahaemolyticus* is not a problem. i.e. not causing diseases to the animal(s) of interest. Under such circumstances, the use of XTS1.2.9 may have an advantage over the use of other bacteria, such *Bacillus* species. This is because, in comparison with other bacteria, *V. parahaemolyticus* is more physiologically and ecologically similar to *V. harveyi* and thus may adapt better to the conditions where the pathogenic *V. harveyi* habits. We have not studied the pathogenicity of XTS1.2.9 but it is known that *V. parahaemolyticus* may be pathogenic to some animals but may not be pathogenic to certain animals where *V. harveyi* can be pathogenic^24^. Furthermore, not all *V. parahaemolyticus* strains are pathogenic to marine animals. Nevertheless, the use of XTS1.2.9, if can be considered, should be applied with much care.

## Methods

### Strains, media and chemicals

Strain NBRC15634 (or *V.h* in short), a type strain of *Vibrio harveyi* provided by NBRC (Japan), was used in most of the tests in this study. Some *V. harveyi* mutants were also used in inhibition mechanism tests. They include^25^: BB886 (*luxPQ*::Tn5 Kan^R^; sensor 1 ( +); sensor 2 (−)), JAF375 (*luxN*::Cm^R^ *luxQ*::Kan^R^; sensor HAI-1 (−); sensor CAI-1 ( +); sensor AI-2 (−)), JMH597 (*luxN*::Tn5 *cqsS*::Cm^R^; sensor HAI-1 (−); sensor CAI-1 (−); sensor AI-2 ( +)) and BB170 (*luxN*::Tn5; sensor HAI-1 (−); sensor CAI-1 ( +); sensor 2 ( +)). These strains were all derived from strain BB120, and thus BB170 is actually a CAI-1 gene complemented strain to JMH597^19,25^. The strains were kindly provided by Bassler Laboratory, Princeton University and McDougald Laboratory, Nanyang Technological University. The strains were cultured and subcultured on the selective thiosulfate-citrate-bile salts-sucrose (TCBS) medium^25^ (Himedia, India) or on Luria-Bertani (LB) medium^26^ containing 3% NaCl, which were predetermined by us (data not shown) to be optimal for the strains to grow. When the strains were solid-cultured, agar was included in the medium (to the final concentration of 2% w/v). The chemicals used for our experiments were purchased from credited suppliers such as Thermo Scientific (USA), Fermentas (USA), Merck (Germany, Xilong (China), Himedia (India), etc.

### Samples

A number of soil and mud samples were taken from Xuan Thuy National Park (mangrove) (Nam Dinh province), Thai Thuy district (Thai Binh province) and some lakes and aquaculture ponds in Ninh Binh province, Vietnam. Sampling was done by following Environmental Protection Agency (EPA) standard protocols (https://www.epa.gov). The samples were used for isolation and screening processes described below.

### Isolation and screening for bacterial strains that inhibit the QS-associated bioluminescence of *Vibrio harveyi*

Isolation was done in the conventional manner based on dilution and spread-plating, by solely using LB medium supplemented with 3% NaCl. The obtained isolates were screened for those quenching the bioluminescence of *V.h* by a 96-well plate assay devised by our group, as described below.

### The 96-well plate assay

*V.h* was incubated while shaken at 150–200 rpm in 3% NaCl LB broth overnight, at 30 °C on a rotary shaker, until the OD_600nm_ of the culture reached approximately 1.0 (normally producing strong luminous intensity). Each isolate was incubated while shaken until the OD_600nm_ of the culture reached approximately 1.0. To test the inhibitory activity of each isolate, a mixture of 240 µl of 3% NaCl LB broth, 10 µl of *V.h* culture broth and 1 µl of the tested isolate culture broth was mixed and filled into a well. The control was a well that only contained 240 µl of 3% NaCl LB broth and 10 µl of *V.h* culture broth. Another control was a well that contained only 3% NaCl LB, which should not luminesce. The 96-well plates containing those experimental mixtures were covered (with their respective lids) and kept stable at 30 °C for 14 h. After that, the plates were observed in the dark. The less bright or dark wells compared with control wells could be suspected to contain the strains that inhibit *V. harveyi* QS-associated bioluminescence. The wells that were normally as bright as the control wells could be judged to contain the strains that did not have the activity. Alternatively, the bioluminescence of the suspected wells were further examined.

To confirm that the inhibitory activity of a strain (such as XTS1.2.9) was not due to growth competition but due to the products in its culture, similar assays were done by mixing the culture broth of *V.h* with: (i) the same volume of the lysed-cell culture broth of the tested strain, (ii) the same volume of the supernatant of the centrifuged culture broth of the tested strain, (iii) the cell pellet of the centrifuged culture broth of the tested strain, and (iv) different amounts of the cell-free culture broth of the tested strain: 5, 10, 25, 50, 100 or 150 µl. The lysed-cell culture broth of the tested strain was prepared by chilling at − 20 °C for 2 h and cell death was checked by plating (data not shown). Centrifugation of the culture broths was carried out at 5000 × g for 15 min. at room temperature. The cell-free culture broth of the strain of interest was prepared by centrifugation as described and filtration using a sterile 0.22 µm filter (Whatman, USA).

### The agar plate assay

This is actually the standard agar diffusion assay for testing antibiotic activities of microorganisms^26^. Thus in brief *V.h* was liquid-cultured until the OD of the culture reached 1.0 before it was spread onto 3% NaCl LB agar (70 µl of the liquid culture per plate). Holes were created in the agar by punching the agar with sterile plastic straws (10 mm in diameter). For a *growth inhibition assay*, right after the spreading, 200 µl of the cell-free culture broth (harvested by centrifugation and sterile filtration) of a tested strain was transferred into a hole while the same amount of sterile 3% NaCl LB broth was filled into another hole as the control. The plates were then kept at 4 °C in 2 h for diffusion of substances in the broths into the agar before incubated at 30 °C for about 14–16 h. After the incubation, if a clear zone of inhibition appeared around the hole, it demonstrated that the suspected strain inhibited the growth of *V.h*. A similar procedure was applied for a *bioluminescence inhibition assay*, except that 500 µl of each tested cell-free culture broth (or 3% NaCl LB broth for the control) was filled into a hole only after the incubation, when the growth of *V.h* was clear, but not right after the spreading. The plates were subsequently observed with time in the dark or analyzed for their luminescence intensities.

### luxR gene expression analysis

The expression of the *luxR* gene, encoding the key regulator protein LuxR that links quorum sensing signaling to bioluminescence, was evaluated by reverse-transcription polymerase chain reaction (RT-PCR)^16^. *V.h* culture broth was prepared as described above before mixed with the same volume of the cell-free culture broth of the tested strain or the same volume of 3% NaCl LB liquid medium (the control). The mixtures were shaken gently and 1 ml of each mixture was taken immediately after mixing (0 h timepoint) and two hours after mixing (2 h timepoint). Total RNA of each taken 1 ml sample was extracted using the SV Total RNA Isolation System kit (Promega, USA). The extracted RNA was used as the template for RT-PCR with the LuxR_Vh_ primer pair (forward: 5′-TCAATTGCAAAGAGACCTCG-3′;

reverse: 5′-AGCAAACACTTCAAGAGCGA-3’) and a thermo cycle (Applied Biosystems 7500 Real-Time PCR System, Thermo Fisher Scientific, USA) as follows^16^: 50 °C—2 min.; 95 °C—10 min.; and 40 cycles of: 95 °C−30 s, 54 °C−1 min., 60 °C−1 min. A final heating step (changing temperature from 60 to 95 °C at 0.1 °C s^-1^ ramping speed) was applied to dissociate the amplicons. Amplicon dissociation curves were determined by constantly measuring fluorescence emission from SYBR™ Green used in the RT-PCR mixtures. Cq values of analyzed samples were calculated by the software MyGo Pro following the guidelines of the manufacture of the real-time PCR machine.

### Taxonomic identification of the *Vibrio* sp. strain of interest

The strain selected after screening was investigated in terms of colony morphology and cell morphology, following standard procedures^26^. As it was suspected to be a *Vibrio* species, it was later subjected to some biochemical tests, including the Methyl Red (MR) test, Voges-Proskauer (VP) test, O-nitrophenyl-β-D-galactopyranosidase (ONPG) test, for further identification according to Jayasinghe et al.^17^. The strain was also identified based on phylogenetic analyses. The 16S rRNA gene fragment of the strain was amplified by colony-PCR, using the primers P63F and P1378R according to Muyzer et al.^27^, and sequenced by First Base (Singapore). The house-keeping gene *recA*^18^ of the strain was also amplified by colony-PCR, using the primers PVbrecA-1F (5’-GAAACCATTTCAACGGGTTC-3’) and PVbrecA-1R (5’- CCATTGTAGCTGTACCAAGCACCC-3’). The 16S rRNA and the *recA* gene sequences of the strain were analyzed to further confirm the taxonomic identification of the strain. The analysis of DNA sequences and homology searches were completed with standard DNA sequencing programs and the BLAST server of the National Center for Biotechnology Information (NCBI) using the BLAST algorithm^28^.

### Tests with the mutants (to explore the QS-associated bioluminescence inhibition mechanism of the strain of interest)

The similar agar plate based bioluminescence inhibition assay was employed to investigate the inhibitory activities of the strain of interest against not only *V.h* but also the mutants, including BB886, JAF375, JMH597 and BB170; except that the Petri plates used for these experiments were only 5 cm in diameter (so that they could fit into the luminometer chamber).

### Image capture and analysis

While observing bioluminescence changes in the dark, photos of 96-well assay plates and agar assay plates were taken with a Canon EOS-7D camera with an aperture time of 30 s. Light intensities of the taken photos were analyzed using ImageJ.

### Luminescence measurement

Bioluminescence intensities from the assay wells or plates were measured by using a luminometer (Turner Designs TD-20/20 Luminometer, Promega, USA). The exposure time for each measurement was 30 s.

### Data analysis

Data were analyzed by using basic statistical methods with tools in Microsoft Excel: differences in data were evaluated by t-Test analysis; errors among replicates were expressed in the form of standard deviations.

Unless otherwise stated, each experiment in this study was done in at least triplicate, i.e. at least 3 wells per assay for 96-well plate assays or 3 plates per assay for agar plate assays.

## Supplementary Information

Supplementary Information.

## Data Availability

Most of the data generated or analysed during this study are included in this manuscript and its supplementary information file. Any other raw data are available from the corresponding author on reasonable request. The sequence data of the 16S rRNA gene and the *recA* gene of strain XTS1.2.9 were deposited in GenBank, under the accession numbers of ON150858 and ON248832, respectively.
