# Supplementary material for: A novel finding of intra-genus inhibition of quorum sensing in Vibrio bacteria
Source: Sci Rep. 2022 Sep 8;12:15203. doi: 10.1038/s41598-022-19424-w (PMC9458646; doi:10.1038/s41598-022-19424-w)
Supplement: Supplementary file 1 — Supplementary Information. [file 41598_2022_19424_MOESM1_ESM.docx]

SUPPLEMENTAL INFORMATION FOR:

**A novel finding of intra-genus inhibition of quorum sensing in *Vibrio* bacteria**

By Huong Thanh Hoang et al.


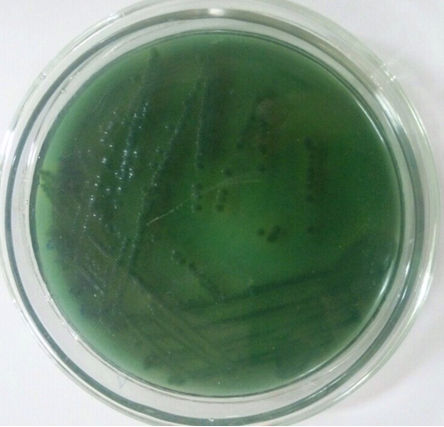

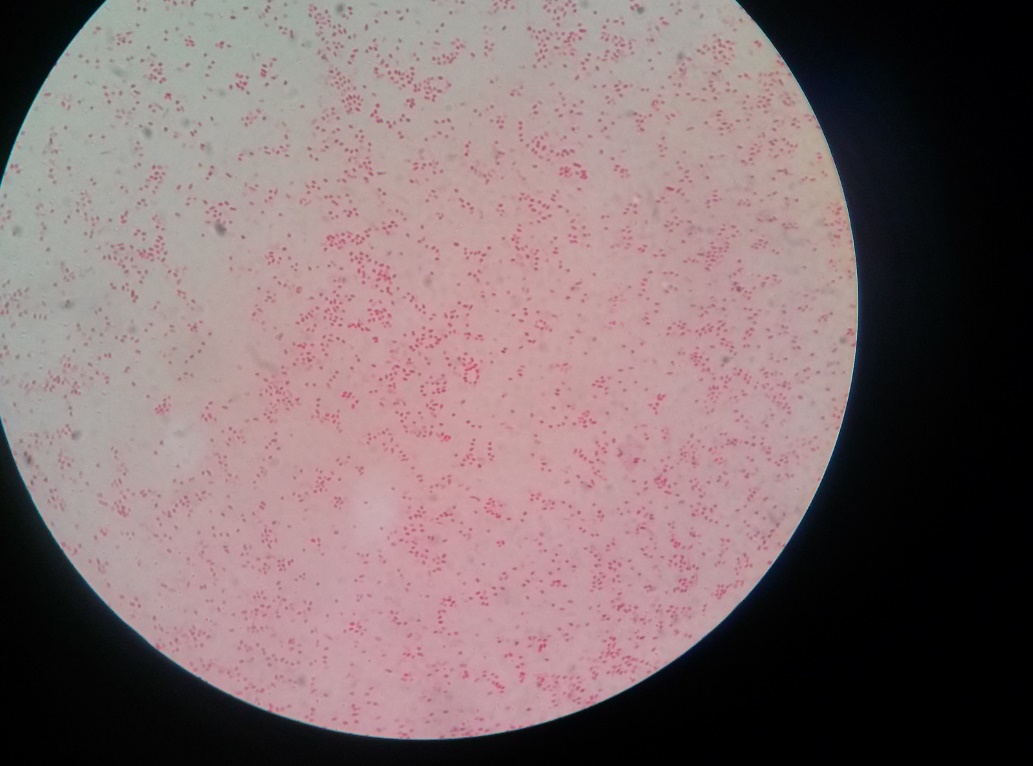


**Figure S1. Morphology of strain XTS1.2.9.** Notes: left: colony morphology on TCBS agar; right: cell morphology observed under a light microscope with a magnification of 1000×.


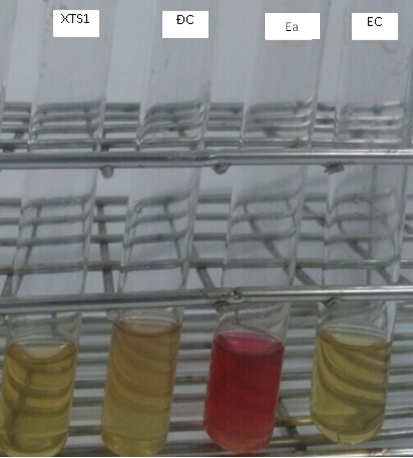


**Figure S2. The MR test result for XTS1.2.9.** Notes: ĐC: control contains only sterile MR medium; XTS1: culture broth of XTS1.2.9; Ec: *E.coli*; Ea: *Enterobacter aerogenes.* Tube(s) with the solution(s) turned red: positive reaction; tube(s) with the solution(s) remaining yellow: negative reaction


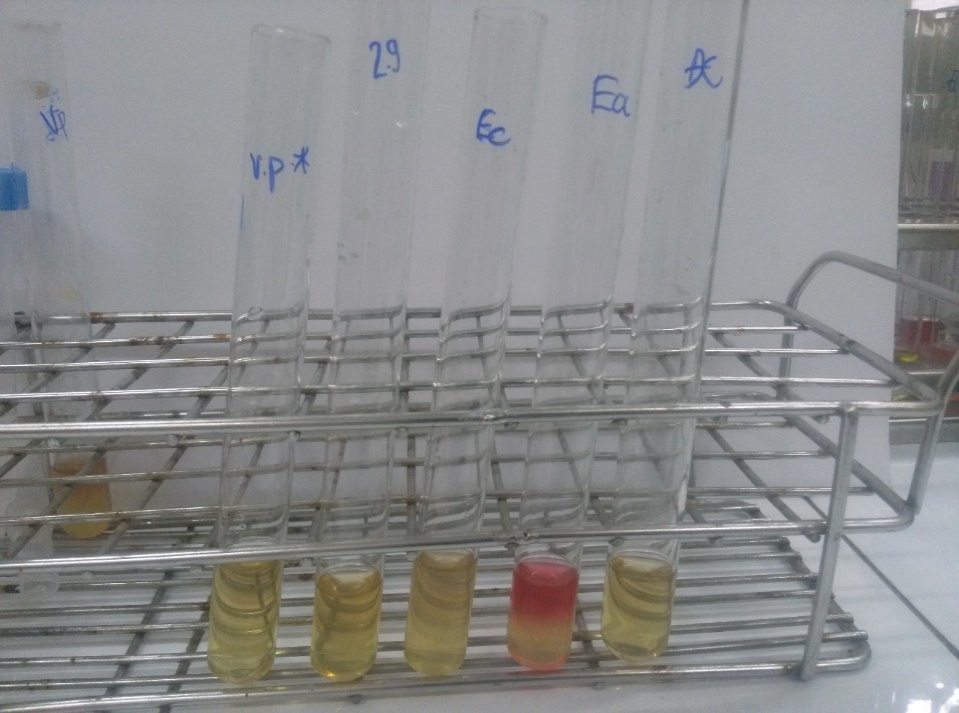


**Figure S3. The VP test result for XTS1.2.9.** Notes: ĐC: control contains only sterile VP medium; 2.9: culture broth of XTS1.2.9; Ec: *E.coli*; Ea: *Enterobacter aerogenes.* Tube(s) with the solution(s) turned red: positive reaction; tube(s) with the solution(s) remaining yellow: negative reaction


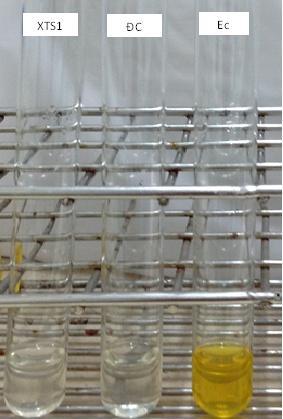


**Figure S4. The ONPG test result for XTS1.2.9.** Notes: ĐC: control contains only sterile ONPG medium; XTS1: culture broth of XTS1.2.9; Ec: *E.coli.* Tube(s) with the solution(s) turned yellow: positive reaction; tube(s) with the solution(s) remaining colorless: negative reaction


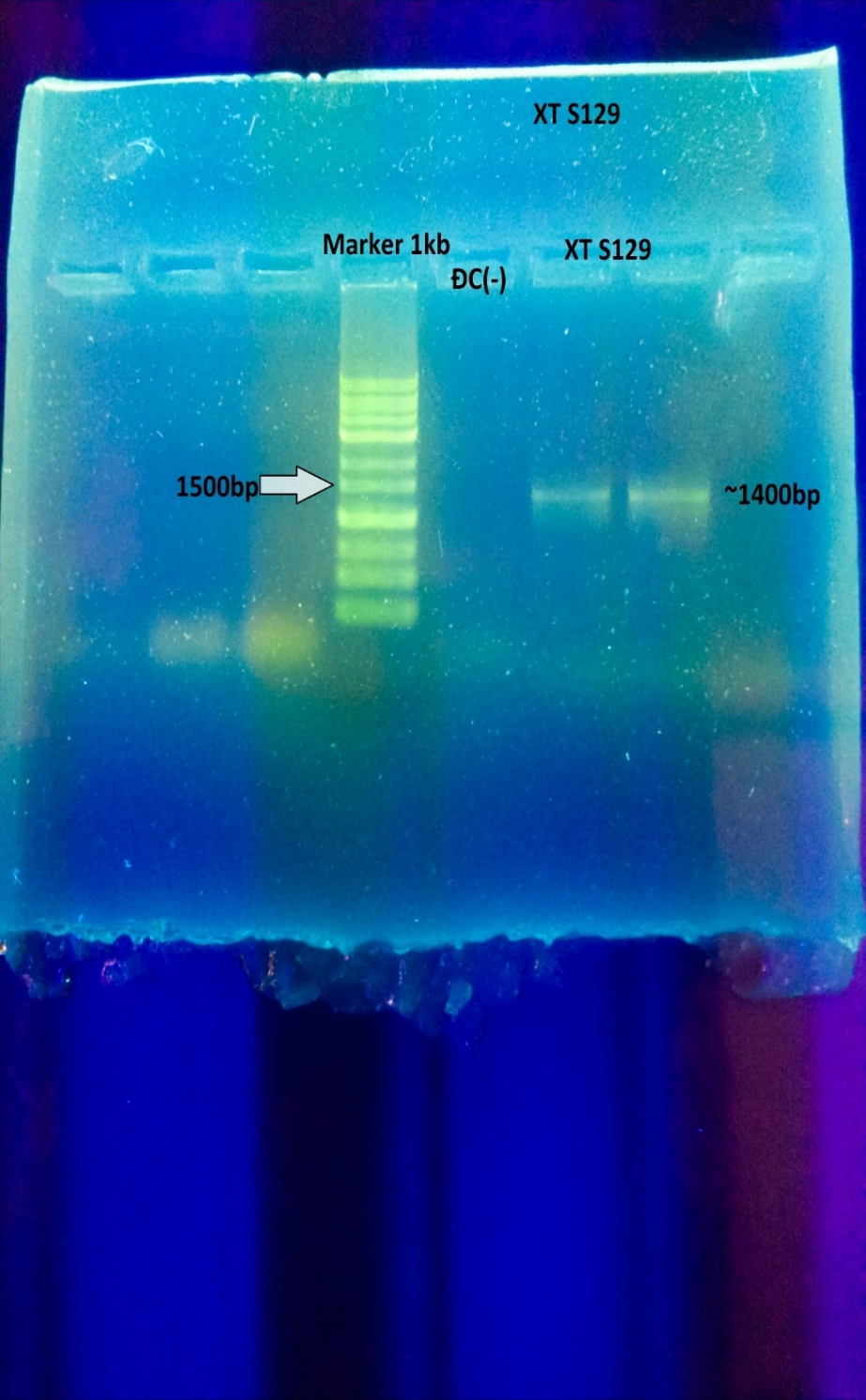


**Figure S5. The result of the electrophoresis to evaluate the PCR to amplify 16S rRNA gene fragement of XTS1.2.9.** Notes: ĐC (-): Negative control; XT S1.2.9: PCR product(s) from XTS1.2.9.


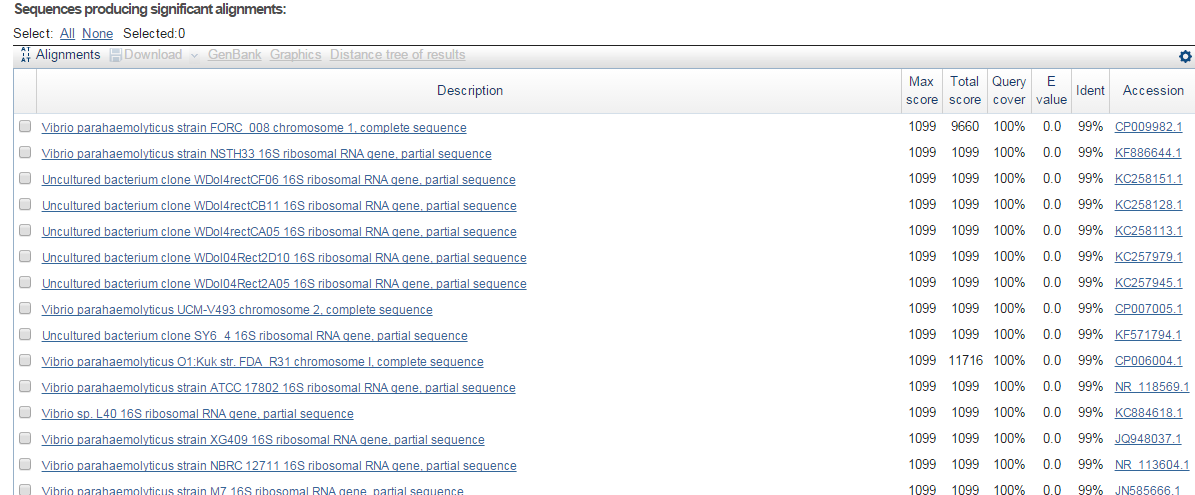


**Figure S6. The result of the BLAST nucleotide sequence analysis of the 16S rRNA gene fragement of XTS1.2.9.**

**
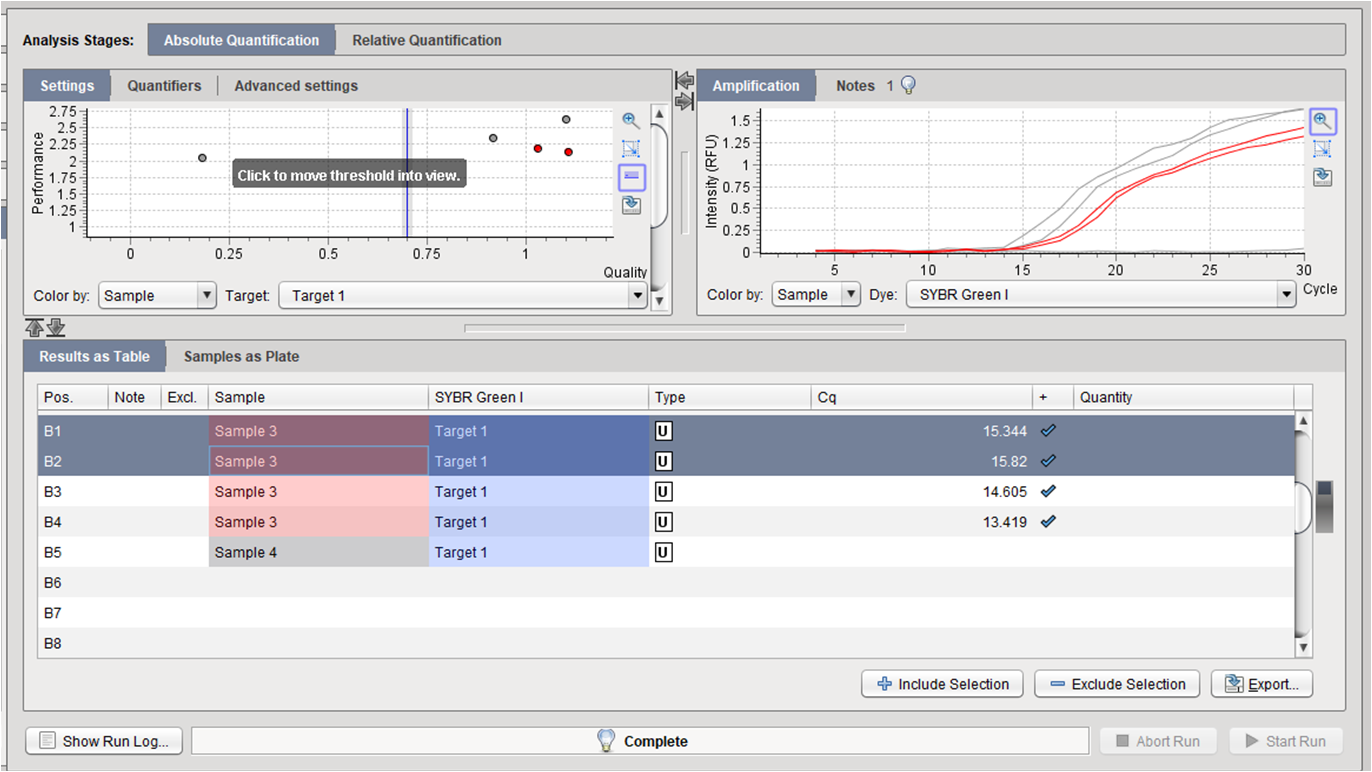
**

**Figure S7. Screen capture picture displaying RT-PCR results.** Sample notes: B1: Vh-X-0h; B2: Vh-X-2h; B3: Vh-C-0h; B4: Vh-C-2h; B5: negative control. (The original raw data file of these results can be found in the following link: <https://drive.google.com/file/d/1BMerYjIlCpuLWbxrBWtYJysAlAs22njc/view?usp=sharing>)
